# Anti-tuberculous drug–induced DRESS syndrome in pregnancy with hepatitis E and autoimmune overlap: A case report

**DOI:** 10.1097/MD.0000000000049747

**Published:** 2026-07-17

**Authors:** Muhammad Tanveer Alam, Syed Muhammad Kashif, Muhammad Luqman, Sandesh Raja, Afeera Bashir, Azzam Ali, Hari Lal, Aayush Chaulagain

**Affiliations:** a Department of Medicine, Dow Medical College, Dow University of Health Sciences, Karachi, Pakistan; b Department of Medicine, Dr. Ruth K.M Pfau Civil Hospital, Karachi, Pakistan; c Department of Medicine, Patan Academy of Health Sciences, Lalitpur, Nepal.

**Keywords:** anti-tuberculous therapy, autoimmune overlap, DRESS syndrome, hepatitis E, pregnancy, tuberculosis

## Abstract

**Rationale:**

Drug reaction with eosinophilia and systemic symptoms (DRESS) is a rare, potentially life-threatening hypersensitivity reaction. Its occurrence during pregnancy, especially secondary to anti-tuberculous therapy (ATT), is exceedingly uncommon and presents major diagnostic and therapeutic challenges.

**Patient concerns:**

A 23-year-old pregnant woman (14 weeks gestation) presented with prolonged fever, jaundice, rash with desquamation, and respiratory symptoms while on first-line ATT for pulmonary tuberculosis.

**Diagnoses:**

Laboratory evaluation revealed severe eosinophilia, deranged liver function, hepatitis E virus co-infection, and autoimmune overlap (systemic lupus erythematosus with antiphospholipid antibody positivity). Based on clinical features and a Registry of Severe Cutaneous Adverse Reactions score of 6, a definite diagnosis of DRESS syndrome was established.

**Interventions:**

ATT was discontinued, and she was treated with systemic corticosteroids, antihistamines, and topical therapy. Sequential drug challenges confirmed hypersensitivity to all 4 first-line agents, necessitating initiation of bedaquiline, clofazimine, and delamanid.

**Outcomes:**

The patient showed favorable clinical recovery with improvement in systemic manifestations and stabilization of pregnancy. She was discharged on modified ATT with multidisciplinary follow-up.

**Lessons:**

This case highlights the complexity of diagnosing and managing DRESS in pregnancy, particularly in tuberculosis-endemic regions. Coexisting viral hepatitis and autoimmune disorders may mimic or exacerbate the syndrome, underlining the importance of high clinical suspicion, timely drug withdrawal, and individualized therapy.

## 1. Introduction

Drug reaction with eosinophilia and systemic symptoms (DRESS) syndrome is a rare, potentially life-threatening, delayed hypersensitivity reaction characterized by fever, widespread cutaneous eruptions, hematologic abnormalities, most notably eosinophilia and multi-organ involvement.^[1]^ It typically manifests within 2 to 8 weeks of exposure to an offending agent and carries a considerable risk of morbidity and mortality, particularly in the presence of hepatic dysfunction.^[2]^

A wide range of medications have been implicated in DRESS syndrome, with anticonvulsants, sulfonamides, and certain antibiotics being the most frequent triggers.^[3]^ Although less common, several first-line anti-tuberculous agents, including isoniazid, rifampicin, ethambutol, and pyrazinamide, have also been reported as potential causative drugs.^[4,5]^ This is of relevance in tuberculosis (TB), which remains a major global health concern, especially in low- and middle-income countries. In 2023, an estimated 10.8 million individuals worldwide developed TB.^[6]^ The disease often necessitates prolonged multidrug regimens, inherently increasing the risk of severe adverse drug reactions such as DRESS syndrome.

Diagnosing DRESS is challenging due to its frequent nonspecific manifestations, which may mimic infections or autoimmune diseases. The Registry of Severe Cutaneous Adverse Reactions scoring system is widely used to aid diagnosis.^[7]^ In pregnancy, these challenges are further compounded by physiological and immunologic alterations and the necessity to carefully balance maternal and fetal safety in therapeutic decision-making. We describe a diagnostically challenging case of a 23-year-old pregnant woman who developed DRESS syndrome following anti-tuberculous therapy (ATT), further complicated by concomitant hepatitis E infection. This case highlights the importance of maintaining a broad differential diagnosis when evaluating systemic drug reactions in high-risk populations. This case report was prepared in accordance with the CARE reporting guidelines and complies with the ethical principles outlined in the Declaration of Helsinki. This case report was reviewed and deemed exempt from formal ethical approval by the Institutional Review Board of Dr. Ruth K.M. Pfau Civil Hospital. Written informed consent was obtained from the patient after she was thoroughly informed about the purpose of this report, possible risks and benefits, and the use of her clinical information for academic publication. She was assured of anonymity and confidentiality and agreed to the publication of this case.

## 2. Case presentation

**Figure 1.**
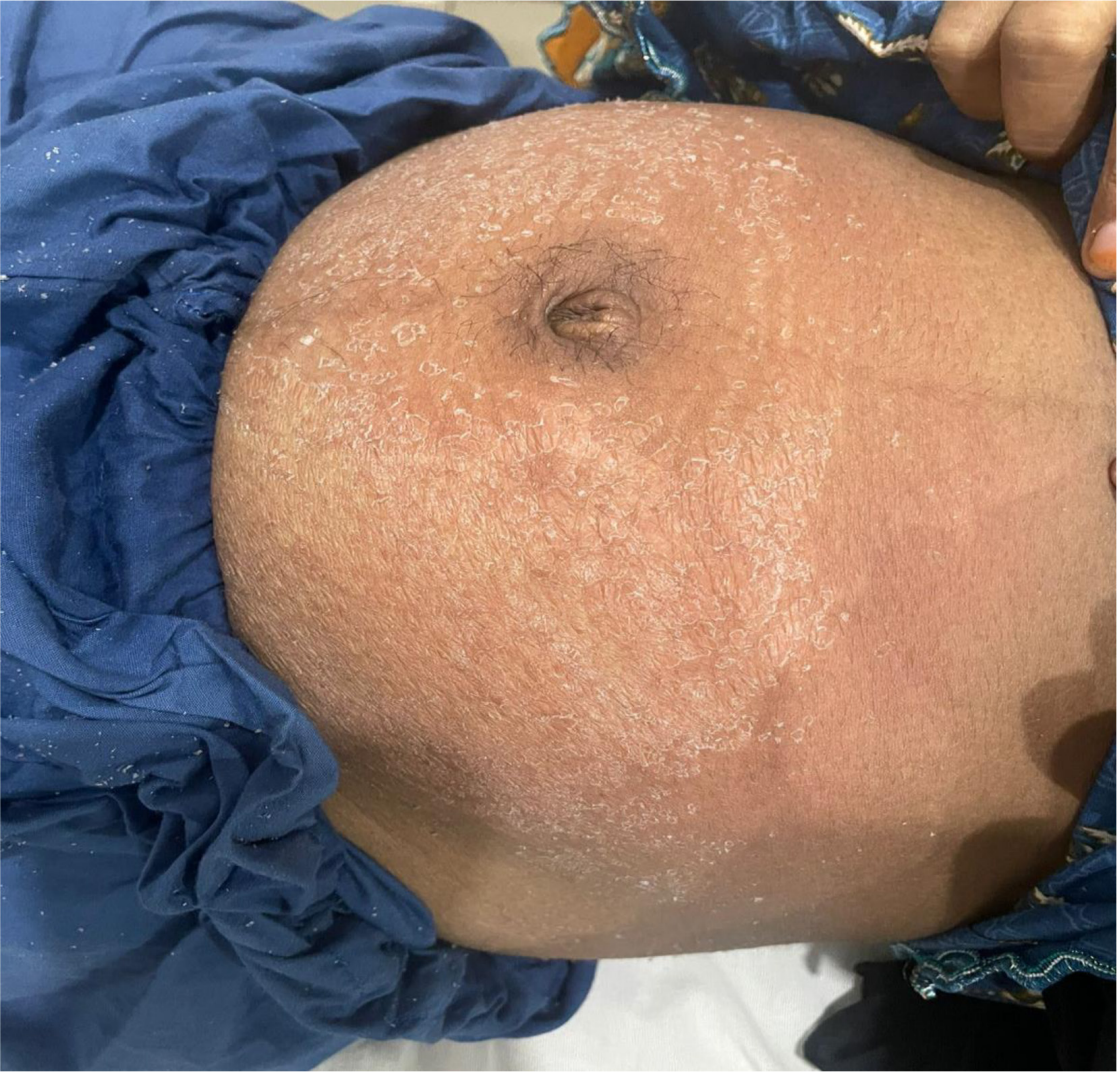
Diffuse erythematous rash with desquamation observed on the abdominal region.

A 23-year-old pregnant woman (gravida 5, para 2, abortions 2 [G5P2 + 2], 14 weeks gestation), with no known comorbidities and a history of areca nut use, presented with 30 days of low-grade intermittent fever, progressive exertional dyspnea, and productive cough, along with 15 days of jaundice, pruritus, diffuse erythematous rash with desquamation (Fig. 1), and nausea. She had been diagnosed with pulmonary tuberculosis 2 months earlier via sputum GeneXpert and was on first-line ATT (ethambutol hydrochloride 275 mg, rifampicin 150 mg, isoniazid 75 mg, pyrazinamide 400 mg) for 5 weeks before presentation. Family history was notable for her father’s death from decompensated chronic liver disease.

On examination, she was alert, afebrile, and hemodynamically stable, with pallor, deep icterus, facial puffiness, periorbital edema, angular cheilitis, oral ulcers, generalized desquamating rash, and restricted mouth opening. Crepitations were present in the right upper lung zone; cardiovascular and abdominal examinations were unremarkable.

Laboratory evaluation demonstrated microcytic hypochromic anemia (hemoglobin 7.7 g/dL, mean corpuscular volume 61 fL) with a reticulocyte count of 4.09%. Iron studies revealed serum iron 114 µg/dL, total iron binding capacity 152 µg/dL, and ferritin 417 ng/mL. High-performance liquid chromatography showed hemoglobin A 94.7%, hemoglobin A2 4.1%, and hemoglobin F 1.2%, consistent with β-thalassemia minor. There was marked leukocytosis (31,000/µL) with severe eosinophilia (71%, absolute eosinophil count 22 × 10^9^/L), platelets 206 × 10^9^/L, prolonged prothrombin time (16 seconds, international normalized ratio 1.5), elevated C-reactive protein (29 mg/dL), lactate dehydrogenase (935 U/L), and deranged liver function tests (total bilirubin 7.1 mg/dL, direct 5.9 mg/dL, alanine aminotransferase 441 U/L, aspartate aminotransferase 200 U/L, alkaline phosphatase 377 IU/L, albumin 2.6 g/dL). Viral serology was negative for hepatitis A, B, C, and human immunodeficiency virus, but reactive for hepatitis E immunoglobulin M and G.

Autoimmune workup revealed a positive direct Coombs test and complement levels (complement component 3 [C3]: 0.8 g/L, complement component 4 [C4]: 0.104 g/L). Antinuclear antibody was positive (cytoplasmic pattern, 1:320) with negative anti-double-stranded deoxyribonucleic acid; in conjunction with the clinical and laboratory features, these findings support a diagnosis of systemic lupus erythematosus^[8]^ (Table 1). Furthermore, persistent anti-β2 glycoprotein I antibody positivity with negative anticardiolipin antibody, along with a history of 2 miscarriages at 10 and 12 weeks’ gestation, fulfills the classification criteria for antiphospholipid antibody syndrome.^[9]^ (Table 2).

**Table 1:** Application of the 2019 EULAR/ACR classification criteria for systemic lupus erythematosus.

Domain/criterion	2019 EULAR/ACR criteria (points)	Patient finding	Score
Entry criterion	ANA ≥1:80 required	ANA positive (1:320)	Yes
Constitutional	Fever (2)	Fever for 30 d	2
Hematologic	Leukopenia (3), thrombocytopenia (4), autoimmune hemolysis (4)	Autoimmune hemolytic anemia (Hb low + Coombs+)	4
Neuropsychiatric	Delirium (2), psychosis (3), seizure (5)	None	0
Mucocutaneous	Oral ulcers (2), nonscarring alopecia (2), acute cutaneous lupus (6), subacute cutaneous OR discoid lupus (4)	Oral ulcers, alopecia	2 + 2 = 4
Serosal	Pleural/pericardial effusion (5), acute pericarditis (6)	SOB + crepitations, but no effusion or pericarditis	0
Musculoskeletal	Joint involvement (6)	None	0
Renal	Proteinuria >0.5 g/d (4), class II–V lupus nephritis (8–10)	None	0
Immunologic: antiphospholipid	Anticardiolipin, anti-β2GPI, lupus anticoagulant (2)	Anti-β2GPI positive	2
Immunologic: complement	Low C3 or low C4 (3), both low (4)	C3 low	3
Immunologic: SLE-specific antibodies	Anti-dsDNA (6), anti-Sm (6)	Both negative	0
Total	15 points (≥10 = SLE)	Definite SLE	

ANA = antinuclear antibody, anti-β2GPI = anti-β2 glycoprotein I antibody, C3 = complement component 3, C4 = complement component 4, dsDNA = double-stranded deoxyribonucleic acid, EULAR/ACR = Application of the 2019 European League Against Rheumatism/American College of Rheumatology, Hb = hemoglobin, SLE = systemic lupus erythematosus.

**Table 2:** Clinical and serological criteria for antiphospholipid antibody syndrome with corresponding patient data.

Domain	APS criteria	Patient’s finding	Meets criterion?
Clinical	≥1 unexplained fetal death of a morphologically normal fetus at ≥10-wk gestation	Miscarriage at 12 wk	Yes
	≥3 consecutive miscarriages <10 wk	1 miscarriage at 10 wk, but not ≥3	No
	Premature birth <34 wk due to eclampsia/placental insufficiency	Not present	No
Laboratory	Lupus anticoagulant positive (2 tests ≥ 12 wk apart)	Not tested/documented	No
	Anticardiolipin IgG/IgM >40 GPL/MPL or >99th percentile (persistent)	Negative	No
	Anti-β2 glycoprotein I IgG/IgM >99th percentile (persistent, ≥12 wk apart)	Positive (persistent)	Yes

APS = antiphospholipid antibody syndrome, GPL = IgG phospholipid units, IgG = immunoglobulin G, IgM = immunoglobulin M, MPL = IgM phospholipid units.

**Figure 2.**
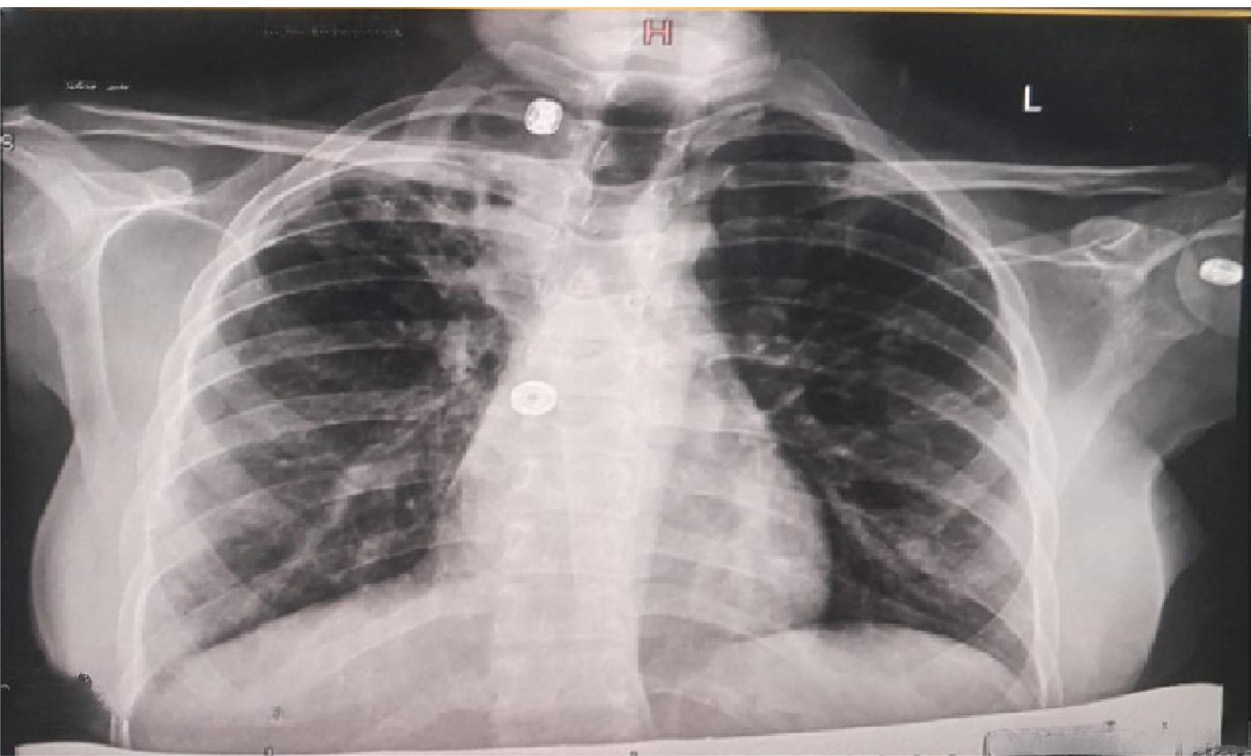
Chest radiograph demonstrating inhomogeneous opacification with cystic lucencies in the right upper lung zone, consistent with bronchiectatic changes.

Abdominal ultrasound showed a normal liver, splenomegaly (15 cm), and a single live intrauterine pregnancy of 13 weeks’ gestation. Echocardiography was unremarkable. Chest radiography revealed inhomogeneous opacification in the right upper lung zone with volume loss and cystic lucencies, consistent with bronchiectatic changes (Fig. 2).

**Figure 3.**
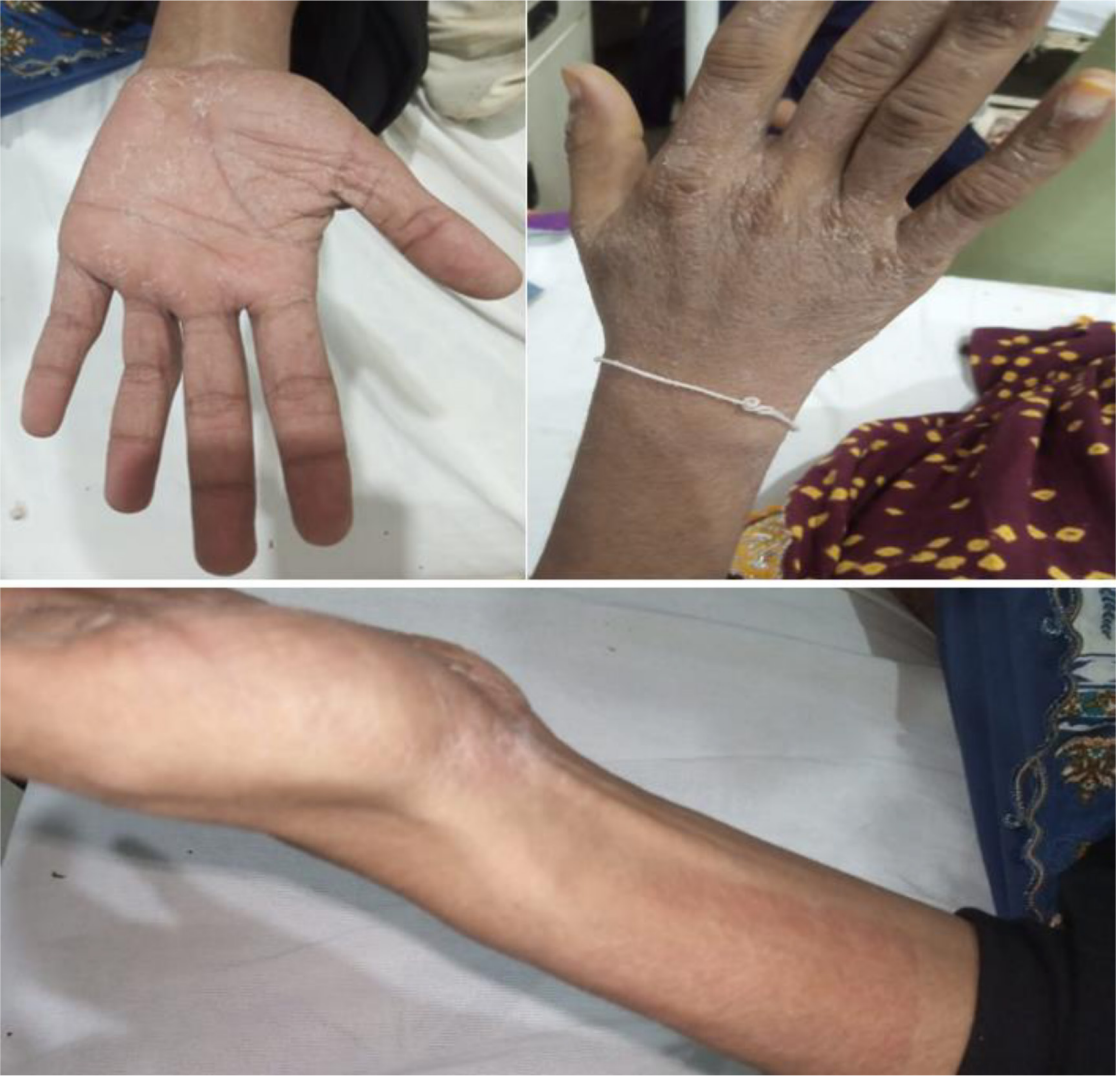
Erythematous rash with desquamation involving the dorsum and palm, noted after anti-tuberculous drug challenge.

Based on clinical features, the patient’s Registry of Severe Cutaneous Adverse Reactions ^[10]^ score for DRESS syndrome was calculated at 6 (Table 3), indicating a definite diagnosis. ATT was discontinued, and she was commenced on intravenous dexamethasone 4 mg twice daily, oral chlorpheniramine 10 mg at bedtime, and topical beclometasone for cutaneous lesions. Clinical stabilization was achieved after 20 days, following which a supervised drug challenge was initiated. Ethambutol was administered first, and within 2 days, the patient developed a rash with peripheral eosinophilia (Fig. 3), managed with intravenous dexamethasone and chlorpheniramine. After a 2-day washout, isoniazid was introduced, leading within 2 hours to shortness of breath, wheezing, and oxygen desaturation; this was treated with intravenous dexamethasone, chlorpheniramine, and nebulized ipratropium. Pyrazinamide challenge induced a rash after 12 hours with eosinophilia, while rifampicin reproduced the rash as well; both episodes were managed with intravenous dexamethasone and chlorpheniramine. These findings confirmed hypersensitivity to all 4 first-line anti-tuberculous agents. The patient was therefore commenced on bedaquiline, clofazimine, and delamanid, to which she showed a favorable response with clinical improvement, and was subsequently discharged. Given the concomitant diagnoses of antiphospholipid antibody syndrome and systemic lupus erythematosus, she was referred to the respective specialty departments for ongoing management.

**Table 3:** Diagnostic criteria for DRESS syndrome according to the RegiSCAR scoring system, applied to the patient’s evaluation.

Domain	Possible values	Patient finding	Score
Fever ≥38.5°C	Yes = +0/no = −1	Fever >38.5°C	0
Enlarged lymph nodes	Yes = +1/no = 0	No lymphadenopathy	0
Eosinophilia	700–1499/µL = +1; ≥1500/µL = +2	Absolute eosinophil count 22 × 10^9^/L	+2
Atypical lymphocytes	Yes = +1/no = 0	None seen	0
Skin involvement >50% BSA	Yes = +1/no = 0	Diffuse erythematous rash with desquamation	+1
Skin rash suggesting DRESS (at least 2: facial edema, infiltrated lesions, desquamation)	Yes = +1/no = 0	Facial puffiness, desquamating erythematous rash	+1
Biopsy suggesting DRESS	Yes = +1/no = 0	Not done	0
Organ involvement	1 organ = +1; ≥2 organs = +2	Lung involvement (SOB + CXR bronchiectasis); spleen (splenomegaly); hematologic (cytopenia); liver uncertain due to hepatitis E	+2
Resolution ≥15 d	Yes = 0/no = −1	Rash >15 d	0
Other potential causes excluded	Yes = +1/no = 0	ANA positive; hepatitis E reactive	0

ANA = antinuclear antibody, DRESS = drug reaction with eosinophilia and systemic symptoms, RegiSCAR = Registry of Severe Cutaneous Adverse Reactions.

## 3. Discussion

DRESS is not only a rare clinical entity but also a condition that challenges the physician’s capacity to balance urgency, precision, and compassion. It is a syndrome that evolves insidiously yet harbors the potential for catastrophic consequences. In this patient, the stakes were heightened as she was young, pregnant, and contending with tuberculosis. Each therapeutic decision carried implications for 2 lives, transforming a medical problem into a profound human dilemma.

The first obstacle lay in diagnostic complexity. Pregnancy inherently modifies the immune system, predisposing women to infections while simultaneously altering hypersensitivity responses.^[11]^ Jaundice in such patients often suggests pregnancy-specific hepatic disorders, including intrahepatic cholestasis or acute fatty liver of pregnancy.^[12]^ The coexistence of hepatitis E infection further complicated the picture, as hepatitis E virus is notorious for precipitating fulminant hepatic failure with disproportionately high maternal mortality.^[13]^ In parallel, the presence of oral ulcers, alopecia, and a history of pregnancy losses raised suspicion for an underlying autoimmune disorder. These overlapping possibilities explain why DRESS is frequently misdiagnosed or recognized only at a late stage, as its presentation closely resembles infections, autoimmune disorders, and systemic complications of pregnancy.^[14]^ Similar diagnostic struggles have been reported in other instances of anti-tuberculosis drug–induced DRESS, where the drug etiology was recognized only after meticulous exclusion of alternative causes.^[15,16]^

The pathophysiology of DRESS underscores its unpredictability. At its core, the syndrome arises from the interaction of toxic drug metabolites with T-cell activation, the release of proinflammatory cytokines, and viral reactivation, most notably of human herpesvirus 6. Genetic predisposition mediated through specific human leukocyte antigen haplotypes further modulates susceptibility, with certain variants heightening the risk of severe drug reactions.^[17,18]^ Recent reviews highlight the synergistic role of latent viral infections and immune dysregulation in precipitating DRESS.^[19]^ Yet, little is known about how these mechanisms intersect with the altered immunologic milieu of pregnancy. This gap in knowledge underscores the need for further research, as pregnancy may both obscure the syndrome’s clinical expression and amplify its severity.

Therapeutic decision-making in this setting was equally fraught. On one hand, drug withdrawal is unequivocally the cornerstone of management.^[20]^ On the other hand, discontinuing anti-tuberculosis therapy in a high-burden context poses grave risks, including maternal mortality, vertical transmission, and adverse fetal outcomes.^[21]^ Corticosteroids provided a path forward in this case, with their utility consistently demonstrated in prior reports for mitigating systemic inflammation and expediting recovery.^[22]^ Reassuringly, evidence suggests that systemic corticosteroids, when judiciously administered, do not compromise tuberculosis control in patients with DRESS.^[23]^ Nonetheless, their use in pregnancy demands careful risk–benefit assessment, balancing potential immunosuppressive effects against the urgency of preventing organ failure.

The question of reintroducing therapy remains among the most difficult challenges in managing DRESS in tuberculosis. Some reports have described graded re-challenge of individual drugs under close monitoring, while others have employed desensitization protocols with success.^[24,25]^ However, recurrence is not uncommon, even with agents structurally unrelated to the original culprits, leaving both patients and clinicians in a state of persistent uncertainty. This uncertainty is not merely clinical but also deeply personal, as patients must grapple with the dual fears of their disease and the very medications prescribed for its treatment.

The literature on anti-tuberculosis drug–induced DRESS during pregnancy remains remarkably sparse. The available report parallels the present case: an initial misdiagnosis, systemic illness characterized by hepatic dysfunction, and subsequent improvement following drug withdrawal and corticosteroid therapy.^[26]^ Though rare, such cases carry consequences of such gravity that vigilance is imperative, particularly in TB-endemic regions where multidrug regimens are routinely prescribed to women of reproductive age.

## 4. Conclusion

This case underscores that managing DRESS requires coordinated, patient-centered care involving hepatology, dermatology, infectious diseases, and obstetrics. Beyond clinical expertise, clinicians must provide reassurance and guidance through the uncertainties of relapse, fetal safety, and treatment response.

## Author contributions

**Conceptualization:** Syed Muhammad Kashif, Muhammad Luqman.

**Supervision:** Muhammad Tanveer Alam.

**Validation:** Muhammad Tanveer Alam, Syed Muhammad Kashif.

**Visualization:** Muhammad Tanveer Alam, Syed Muhammad Kashif.

**Writing – original draft:** Muhammad Luqman, Sandesh Raja, Afeera Bashir, Azzam Ali, Hari Lal, Aayush Chaulagain.
